# Development of Prediction Models Using Machine Learning Algorithms for Girls with Suspected Central Precocious Puberty: Retrospective Study

**DOI:** 10.2196/11728

**Published:** 2019-02-12

**Authors:** Liyan Pan, Guangjian Liu, Xiaojian Mao, Huixian Li, Jiexin Zhang, Huiying Liang, Xiuzhen Li

**Affiliations:** 1 Institute of Pediatrics Guangzhou Women and Children's Medical Center Guangzhou Medical University Guangzhou China; 2 Department of Genetics and Endocrinology Guangzhou Women and Children’s Medical Center Guangzhou Medical University Guangzhou China

**Keywords:** central precocious puberty, GnRHa-stimulation test, machine learning, prediction model

## Abstract

**Background:**

Central precocious puberty (CPP) in girls seriously affects their physical and mental development in childhood. The method of diagnosis—gonadotropin-releasing hormone (GnRH)–stimulation test or GnRH analogue (GnRHa)–stimulation test—is expensive and makes patients uncomfortable due to the need for repeated blood sampling.

**Objective:**

We aimed to combine multiple CPP–related features and construct machine learning models to predict response to the GnRHa-stimulation test.

**Methods:**

In this retrospective study, we analyzed clinical and laboratory data of 1757 girls who underwent a GnRHa test in order to develop XGBoost and random forest classifiers for prediction of response to the GnRHa test. The local interpretable model-agnostic explanations (LIME) algorithm was used with the black-box classifiers to increase their interpretability. We measured sensitivity, specificity, and area under receiver operating characteristic (AUC) of the models.

**Results:**

Both the XGBoost and random forest models achieved good performance in distinguishing between positive and negative responses, with the AUC ranging from 0.88 to 0.90, sensitivity ranging from 77.91% to 77.94%, and specificity ranging from 84.32% to 87.66%. Basal serum luteinizing hormone, follicle-stimulating hormone, and insulin-like growth factor-I levels were found to be the three most important factors. In the interpretable models of LIME, the abovementioned variables made high contributions to the prediction probability.

**Conclusions:**

The prediction models we developed can help diagnose CPP and may be used as a prescreening tool before the GnRHa-stimulation test.

## Introduction

Precocious puberty is related to the development of secondary sexual characteristics in girls before the age of 8 years and in boys before the age of 9 years. In recent years, the age of puberty onset has shown a decreasing trend, and puberty is related to subsequent health outcomes such as breast cancer, diabetes, and behavioral disorders [1]. Central precocious puberty (CPP), also known as true precocious puberty, is caused by early activation of the hypothalamic-pituitary-gonadal axis with clinical pubertal symptoms. CPP can influence final adult height and result in psychological problems, which will cause inappropriate behaviors. It is important for girls with suspected CPP to be evaluated and diagnosed in a timely manner.

The gold standard in the confirmation of CPP is the positive response of gonadotropin to a gonadotropin-releasing hormone (GnRH)–stimulation test. In the absence of GnRH, GnRH analogues (GnRHa) are usually used instead [2]. However, the stimulation test is time consuming and expensive. Besides, the test is painful and make patients uncomfortable due to the need for repeated blood sampling at different time points. Therefore, another method to avoid the disadvantages of the GnRHa-stimulation test will be of great help in the diagnosis of CPP.

Several studies have focused on investigating a single gonadotropin biomarker to identify patients with CPP conveniently. Basal or peak serum luteinizing hormone (LH), follicle-stimulation hormone (FSH), and the ratio LH/FSH are the most common biomarkers reported [3-7]. However, the cut-off values of these single biomarkers depend on the assay used to measure the gonadotropin levels. As a result, cut-off points in previous studies differed widely. Moreover, both the Pasternak group [3] and the Mogensen group [8] reported that a single basal serum LH measurement could verify the presence of CPP, but could not confirm the absence of CPP. Therefore, a single gonadotropin parameter alone may not be sufficient for the diagnosis of CPP, and clinical and laboratory factors that can predict response to the GnRHa-stimulation test should be combined [9,10]. Suh et al [10] found that accelerated growth rate, advanced bone age, and increased basal gonadotropin and insulin-like growth factor-I (IGF-I) levels were correlated with CPP. Traditional statistical analysis including *t* test and binary logistic regression were used to select factors correlated with the GnRH test [11-14]. Although remarkable progress has been made in these studies, there is a long way to go for their application in clinics due to the low sensitivity or specificity of tests.

Considering the previous studies and the extensive application of machine learning algorithms in the medical field, we aimed to determine whether combining multiple variables with machine learning classifiers could improve prediction of the GnRHa-stimulation test and thus help diagnose CPP.

## Methods

### Population and Variables

We enrolled 1757 girls with CPP symptoms who visited the Pediatric Day Ward of the Endocrinology Department at Guangzhou Women and Children’s Medical Center from January 2012 to March 2018. All subjects had undergone the GnRHa-stimulation test. Girls with any other disorders or intracranial lesions were excluded from the study.

Girls fulfilling the following eligibility criteria were considered to have a positive response to the GnRHa-stimulation test and were diagnosed with CPP in our study: (1) peak LH level ≥ 10 IU/L or peak LH level ≥ 5 IU/L combined with a ratio of peak LH to FSH value ≥ 0.6 and (2) onset of secondary sexual characteristics at the age < 8 years. Girls whose laboratory tests did not satisfy all the abovementioned criteria were considered to have a negative response. According to the long-term clinical practices, the first condition with a peak LH ≥ 10 IU/L is used as the diagnosing criterion in our hospital. Peak LH level ≥ 5 IU/L combined with a ratio of peak LH to FSH value ≥ 0.6 is widely used in China and some other countries for children undergoing the GnRH-stimulation test [15,16]. Since the stimulation effect of GnRHa is almost hundreds times that of GnRH [17], a condition that affects the levels of sex hormones due to GnRH would do the same with GnRHa. Our diagnostic criteria are an improved version of the existing criteria that are adapted to our population.

Information such as chief complaints, development of secondary sexual characters, and abnormal duration of puberty were stored as free text in the clinical records of the electronic medical records system. Laboratory test values were reported as structured data in the laboratory information system. In total, 19 variables were extracted from the clinical records and laboratory results for all the 1757 patients. Specifically, 10 variables extracted from the clinical records were weight, height, body mass index (BMI), abnormal duration of puberty in records (History), menarche, core in breast (Core), pigmentation, development stage of pubes (Pubes), development stage of left breast, and development stage of right breast. Breast and pubic hair development were evaluated using Tanner staging (stages 1 to 5). Nine variables extracted from the laboratory results were age, basal serum LH, FSH, estradiol, prolactin, testosterone, growth hormone, IGF-I, IGF-binding protein-3 levels before the GnRHa test.

Among the 1757 patients, 436 girls had examination reports available, including pelvic ultrasonography (for development of the uterus and ovaries) and radiography of the left hand (for bone age). Six variables extracted from the examination reports were development of uterus, existence of follicle, uterine volume, left and right ovary volumes, and bone age. Bone age was measured by the Greulich and Pyle method [18]. The variables from the clinical records and the examination reports were extracted first with traditional regex match using Python [19] and then examined manually by two endocrinologists.

This study was approved by the Institutional Review Board of Guangzhou Women and Children’s Medical Center and conducted in accordance with the ethical guidelines of the Declaration of Helsinki of the World Medical Association. The requirement to obtain informed consent was waived because of the retrospective nature of the study. Data used in this study were anonymous, and no identifiable personal data of the patients were available for the analysis.

### Data Preprocessing

Variables with more than 20% missing data, such as growth rate of height and weight and heights of parents, were excluded from this study. Missing values for continuous variables were replaced with mean values of all the samples grouped by age. Discrete variables like experience of menarche were filled with a value of 0. Discrete variables like Tanner stage for breast and pubes were filled with the least degree (stage 1).

### Model Development and Assessment

Tree learning classifiers allow nonlinear interactions between features and have good interpretability. Considering this, we selected two tree-based ensemble binary classification algorithms—extreme gradient boosting (XGBoost) and random forest—to develop our models. We also used linear supported vector machines (SVM) and decision trees for the classification to compare the performance between ensemble models and nonensemble models. The models aimed to identify relationships between the input features and the output GnRHa test results, thereby determining whether a patient responds positively to the GnRHa test.

XGBoost is a scalable tree boosting and effective learning algorithm [20]. It trains a sequence of models to minimize errors made by existing models. Models in XGBoost are decision trees. Many data scientists have applied this algorithm to solve classification problems and achieved excellent results. XGBoost has also been successfully used in medical studies [21,22]. As XGBoost is essentially a gradient boosting tree model, which is not based on distance, normalization is not required. Random forest is another classical ensemble learning algorithm with a combination of a large amount of trees [23], which trains decision trees in parallel by using data with replacement. It applies bootstrap aggregating to tree learners, which leads to better performance as variance decreases. Random forest has the ability to handle nonlinear data and is robust to noise. Besides, parameter tuning is not that complex for these two algorithms compared to other ensemble learning algorithms. SVM is a binary classifier with a maximum margin hyperplane [24]. The decision tree classifier is a tree-like model used for classification [25].

**Figure 1:**
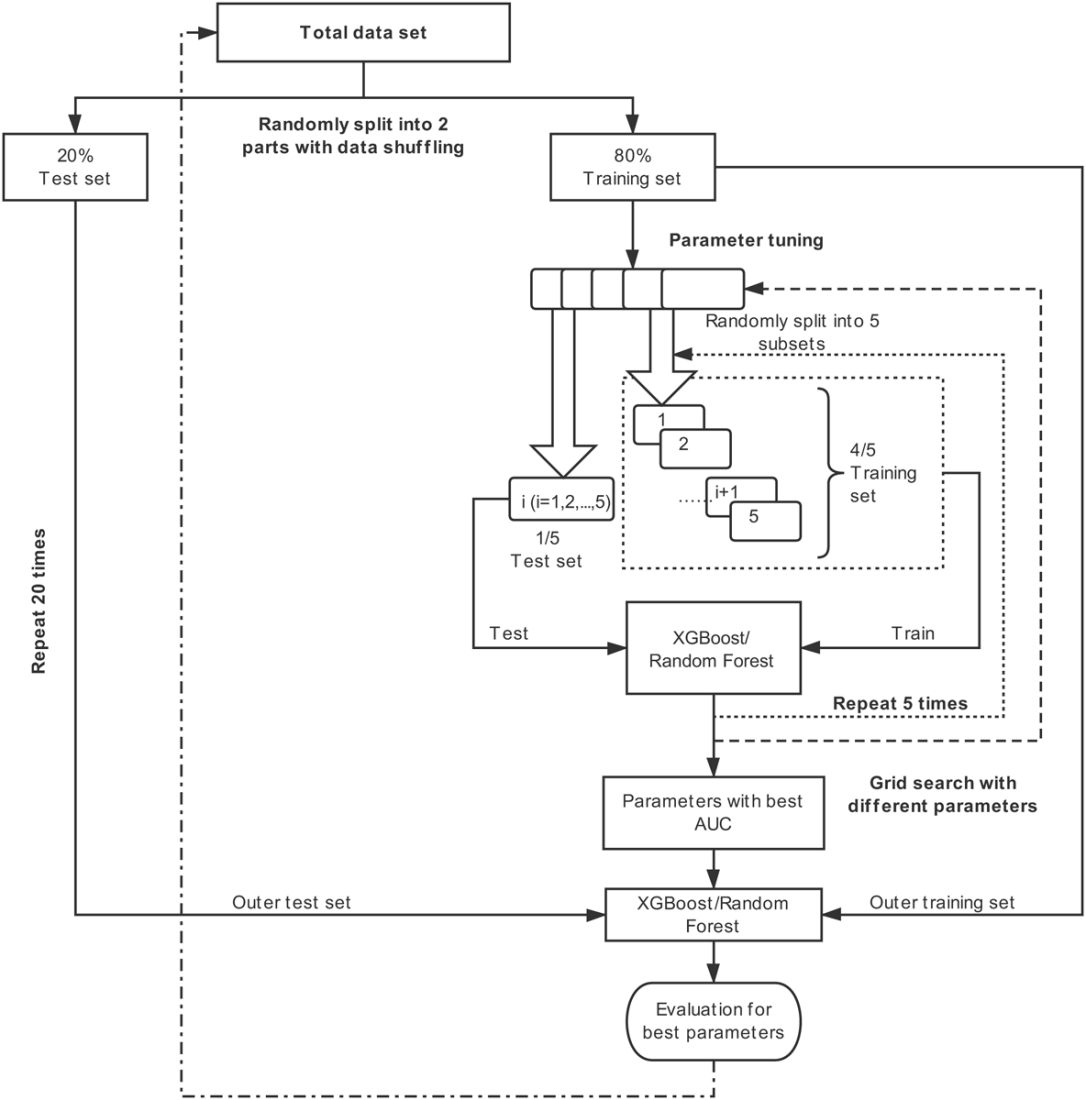
Training and validation process of prediction models. AUC: area under receiver operating characteristic; XGBoost: extreme gradient boosting.

In order to obtain robust assessments and prevent overfitting, we used a nested cross-validation with an outer Monte Carlo cross-validation [26] (MCCV, repeated 20 times) and an inner k-fold cross-validation (k=5) for parameter tuning, yielding a total of 20 times the five-fold cross-validation. In the outer MCCV loop, the whole data set is randomly divided into the training set (80%) and the test set (20%) for 20 times. For each training set, the inner stratified five-fold cross-validation loop is performed as follows. The training set is split into five subsets, where four subsets are used for training and one is used for test. Parameter tuning is performed with grid search in the inner cross-validation. Finally, a model fitted on the training set with parameters that has the best area under the curve (AUC) evaluated on the inner test set is determined. The detailed training and test process is presented in Figure 1.

### Feature Importance

XGBoost and random forest classifier have the ability to evaluate the importance of features. Feature importance is a feature weight and can represent the contribution to prediction. In XGBoost, feature importance is computed by the sum of times that the feature is selected as a tree node. In random forest, feature importance is calculated based on the out-of-bag (OOB) error. OOB error is the mean prediction error for training observations in the respective bootstrap sample. After randomly adding noise perturbations to OOB samples, a feature with a higher OOB error difference is more important, with higher feature importance. For both models, each feature obtained 20 feature importance values with 20 times the MCCV. We summed all the feature importance values for each feature and obtained a rank for all features.

### Model Interpretation

One disadvantage of machine learning is that the model usually runs as a black box. However, it is necessary for a doctor to understand the reasons why a model makes such a prediction in the clinic, especially when timely detection is necessary. Tree-based models can provide feature importance at a global level but not in a specific case. The local interpretable model-agnostic explanations (LIME) algorithm is developed to identify an interpretable model that is locally faithful for each individual prediction [27,28]. It provides relative feature contributions for a single instance of the prediction result. LIME generates neighborhood data by randomly perturbing features from the instance. It then learns locally weighted linear models on this neighborhood data to explain each of the classes in an interpretable way. Parameters in LIME mainly include the maximum number of features in explanation, number of neighborhood samples to generate, and machine learning prediction function. We used the LIME library from the original authors for the model interpretation. The number of neighborhood samples is 5000 by default. The parameter num_features (maximum number of features) is the number of features shown in the explanation. The default value is 10. In our study, the value was 9 for a clear layout, as the contributions of features ranked after 9 were almost zero.

In our study, we used a submodular pick [27] instead of a random pick to select a diverse, representative set of samples from the test set for nonredundant explanations. For these samples, we then obtained the class probabilities, and the representative individuals were assigned with and average weight contributed by each feature to display how the classifier made a decision. Finally, we went over all these LIME results together with the endocrinologists to decide whether we should trust the results of the model.

All computation and visualization were performed in Python [19] using packages like Scikit-learn, Pandas, Lime, and Matplotlib.

## Results

### Subject Characteristics

Among the 1757 girls included in our study, 966 were positive for the GnRHa-stimulation test and diagnosed with CPP; the remaining 791 girls showed a negative response to the test. As shown in Table 1, 16 of the 19 variables were significantly higher in the CPP group than in the non-CPP group (*P*<.05), whereas prolactin, BMI, and pigmentation were similar in both groups.

### Evaluation for Models

**Figure 2:**
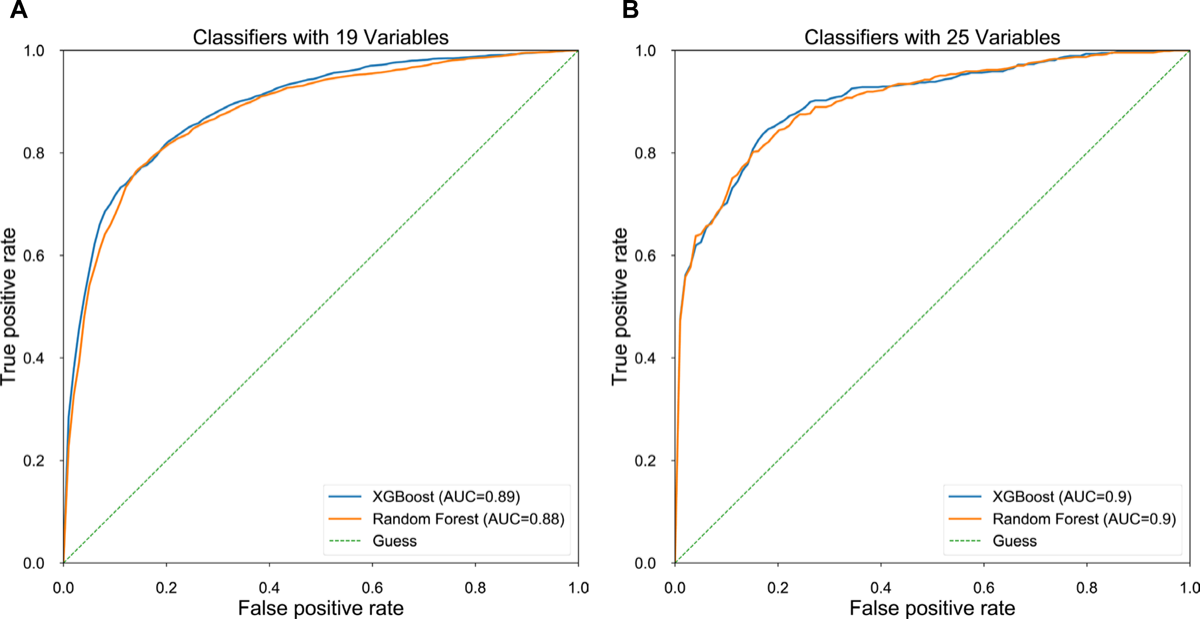
ROC curves for classifiers with 19 variables for 1757 patients and 25 variables for 436 patients. ROC: receiver operating curve; AUC: area under ROC.

First, we developed prediction models with the data of 19 clinical and laboratory variables (Table 1) from all 1757 patients. Two machine learning algorithms, XGBoost and random forest classifiers, were used, and parameters with the best AUC were selected for each model. The performance as well as the selected parameters of the models are listed in Table 2, and their receiver operating characteristic (ROC) curves are plotted in Figure 2. The performance was evaluated on the 20 test sets split with MCCV. Both models had strong prediction powers, with a specificity of ≥84.32%, a sensitivity of ≥77.91%, and an AUC of ≥0.88. The XGBoost classifier is slightly more effective than the random forest classifier, especially in terms of the specificity (*P*<.01), whereas random forest is much more efficient in terms of the computation speed with less model complexity.

**Table 1:** Basic characteristics of girls who underwent the GnRHa-stimulation test.

Variables	Non-CPP^a^ (n=966), mean (SD)	CPP (n=791), mean (SD)	*P* value^b^
Age (years)	7.07 (1.11)	7.52 (0.99)	<.001
LH^c^ (IU/L)	0.12 (0.23)	0.93 (1.28)	<.001
FSH^d^ (IU/L)	1.82 (1.30)	3.01 (1.62)	<.001
GH^e^ (ng/mL)	3.27 (3.26)	4.75 (4.69)	<.001
IGF-I^f^ (ng/mL)	231.35 (65.93)	317.87 (89.84)	<.001
IGFBP-3^g^ (μg/mL)	4.55 (0.52)	4.81 (0.55)	<.001
Estradiol (pmol/L)	102.56 (50.96)	125.81 (60.97)	<.001
Prolactin (ng/mL)	8.73 (5.39)	8.59 (5.61)	.52
Testosterone (nmol/L)	0.80 (0.39)	0.94 (0.49)	<.001
History^h^ (months)	7.67 (10.39)	9.27 (9.63)	<.001
Menstruation/menarche (yes, no)	N/A^i^	N/A	.03
Height^j^ (cm)	127.16 (8.61)	131.61 (8.42)	<.001
Weight^j^ (kg)	27.32 (5.32)	29.60 (4.95)	<.001
BMI^k^ (kg/m^2^)	16.73 (2.30)	16.91 (1.96)	.34
Breast core (yes, no)	N/A	N/A	.02
Pubes^l^ (1-5)	1.06 (0.27)	1.14 (0.44)	<.001
Pigmentation (yes, no)	N/A	N/A	.87
Left breast^l^ (1-5)	2.33 (0.84)	2.76 (0.92)	<.001
Right breast^l^ (1-5)	2.32 (0.84)	2.78 (0.92)	<.001

^a^CPP: central precocious puberty.

^b^The equality of each indicator was evaluated by Chi-square or Student  *t* test. *P*<.05 was considered significant.

^c^LH: luteinizing hormone.

^d^FSH: follicle-stimulation hormone.

^e^GH: growth hormone.

^f^IGF-I: insulin-like growth factor-I.

^g^IGFBP-3: insulin-like growth factor binding protein-3.

^h^Abnormal duration in records.

^i^N/A: not applicable.

^j^At stimulation test.

^k^BMI: body mass index.

^l^Tanner stage.

**Table 2:** Predictive performance of classifiers and the corresponding parameters. A paired *t* test was performed on specificity and sensitivity for comparison against XGBoost.

Algorithms/Variables		Specificity^a^ (%), mean (SD)	Sensitivity^b^ (%), mean (SD)	AUC^c^, mean (SD)	Parameters
**19 variables, 1757 patients**					
	XGBoost^d^	85.39 (1.38)	77.94 (3.50)	0.89 (0.02)	Learning rate=0.01, max depth=3, number of trees=500
	Random forest	84.32 (1.88)^e^	77.91 (3.59)^f^	0.88 (0.02)	Max depth=3, criterion=gini, number of trees=20
	SVM^g^	88.94 (1.76)^e^	62.36 (4.12)^e^	0.86 (0.04)	Kernel=linear, penalty coefficient=5
	Decision tree	75.90 (2.47)^e^	71.71 (3.99)^e^	0.74 (0.02)	Criterion=entropy
**19 variables, 436 patients**					
	XGBoost	83.17 (5.29)	75.28 (6.43)	0.86 (0.04)	Learning rate=0.01, max depth=3, number of trees=500
	Random forest	83.46 (6.28)^f^	74.72 (6.43)^f^	0.85 (0.04)	Max depth=3, criterion=gini, number of trees=20
	SVM	88.94 (4.90)^e^	62.36 (7.73)^e^	0.86 (0.02)	Kernel=linear, penalty coefficient=5
	Decision tree	76.25 (7.07)^e^	68.06 (7.12)^e^	0.72 (0.04)	Criterion=entropy
**25 variables, 436 patients**					
	XGBoost^d^	87.66 (5.52)	76.64 (6.51)	0.90 (0.04)	Learning rate=0.01, max depth=4, number of trees=500
	Random forest	87.41 (4.22)^f^	75.03 (7.91)^f^	0.90 (0.05)	Max depth=3, criterion=entropy, number of trees=20
	SVM	89.81 (4.28)^f^	66.53 (7.01)^e^	0.86 (0.02)	Kernel=linear, penalty coefficient=5
	Decision tree	76.35 (5.51)^e^	68.61 (7.16)^e^	0.72 (0.05)	Criterion=entropy
**1-3 variables, 1757 patients, XGBoost^d^**					
	LH^h^, IGF-I^i^, FSH^j^	83.17 (1.62)	76.39 (3.57)	0.86 (0.02)	Learning rate=0.01, max depth=3, number of trees=500
	LH^h^, IGF-I^i^	83.27 (1.62)	75.69 (3.61)	0.86 (0.02)	Learning rate=0.01, max depth=3, number of trees=500
	LH^h^, FSH^j^	83.56 (1.94)	75.83 (3.13)	0.84 (0.02)	Learning rate=0.01, max depth=3, number of trees=500
	LH^h^	83.37 (2.00)	75.97 (3.74)	0.84 (0.02)	Learning rate=0.01, max depth=3, number of trees=500
	IGF-I^i^, FSH^j^	80.77 (2.47)	57.08 (3.29)	0.77 (0.02)	Learning rate=0.01, max depth=3, number of trees=500
	IGF-I^i^	80.19 (3.14)	53.19 (4.55)	0.73 (0.02)	Learning rate=0.01, max depth=3, number of trees=500
	FSH^j^	84.13 (3.87)	45.00 (5.34)	0.68 (0.02)	Learning rate=0.01, max depth=3, number of trees=500

^a^Specificity=number of true negatives/(number of true negatives+number of false positives).

^b^Sensitivity=number of true positives/(number of true positives+number of false negatives).

^c^AUC, area under the receiver operating curve.

^d^XGBoost: extreme gradient boosting.

^e^*P*<.01

^f^Not significant.

^g^SVM: supported vector machines.

^h^LH: luteinizing hormone.

^i^IGF-I: insulin-like growth factor-I.

^j^FSH: follicle-stimulation hormone.

In the data set, 436 girls had additional examination reports, and we extracted six variables from these reports (see Population and Variables subsection). To investigate whether adding image features could enhance the prediction efficiency, we combined the six variables with the 19 variables and trained and evaluated both the XGBoost and random forest models on the 436 samples, of which 180 patients belonged to the CPP group and 256 belonged to the non-CPP group. For the ease of comparison, we retrained the previous 19-variable models with the 436 samples. As shown in Table 2, the reduction in sample size led to a serious decline in model performance, whereas the addition of six image features improved their performance. Specifically, for XGBoost in 436 samples, the specificity increased from 83.17% for 19 variables to 87.66% for 25 variables, the sensitivity increased from 75.28% to 76.64%, and the AUC increased from 0.86 to 0.90. For random forest in 436 samples with 25 variables, the specificity increased from 83.46% to 87.41%, the sensitivity increased from 74.72% to 75.03%, and the AUC increased from 0.86 to 0.90 compared to the results from 436 samples with 19 variables. Similarly, as seen in the ROC curves shown in Figure 2, XGBoost performed slightly better than the random forest classifier.

To compare performance between ensemble models and nonensemble models, the SVM and decision tree classifiers were used to develop predictive models for the abovementioned settings. Higher specificities were achieved with the SVM models. However, sensitivities for the SVM models were much lower than those for the ensembles models. The decision tree models demonstrated significantly inferior performance in terms of almost all the sensitivities, specificities, and AUCs. These results suggest that the ensemble models are able to yield excellent performance while maintaining a good balance between sensitivity and specificity in the prediction of CPP.

### Feature Importance

**Figure 3:**
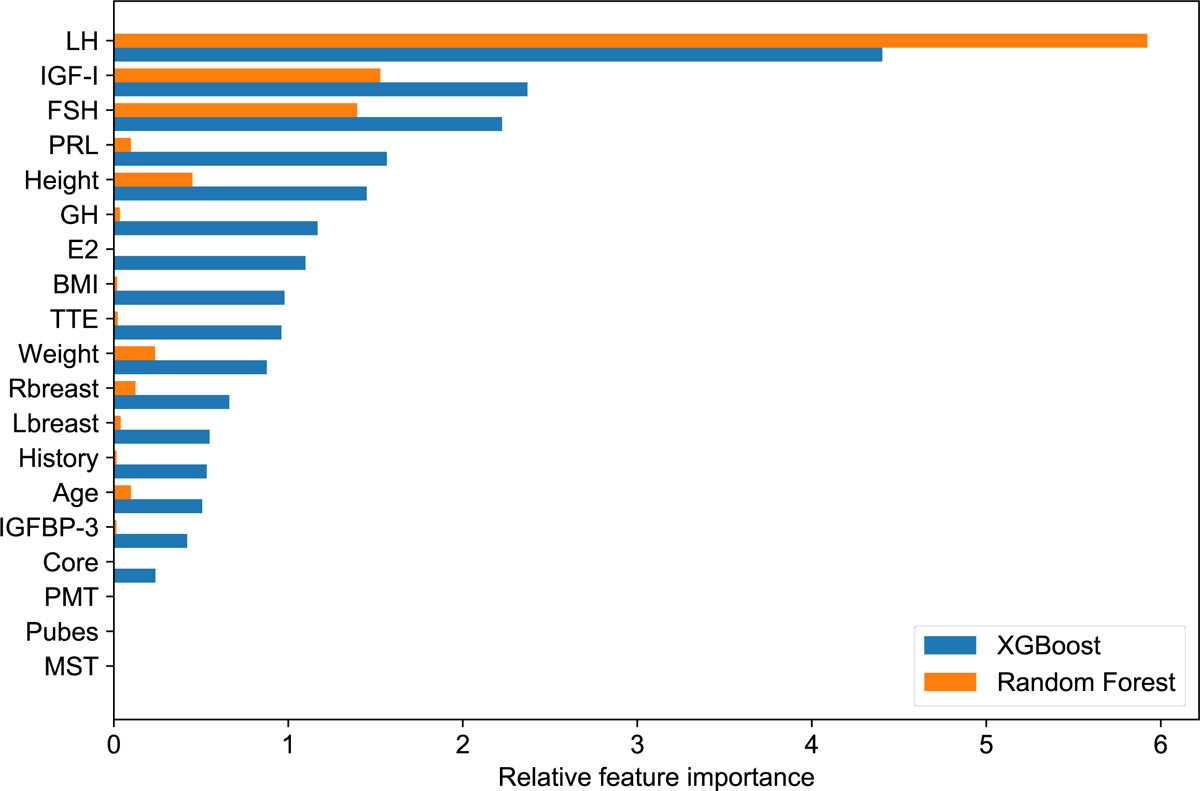
Feature importance ranking for 19 variables in two classifiers calculated by the models. LH: luteinizing hormone; IGF-I: insulin-like growth factor-I; FSH: follicle-stimulation hormone; PRL: prolactin; GH: growth hormone; E2: estradiol; BMI: body mass index; TTE: testosterone; Rbreast: right breast; Lbreast: left breast; IGFBP-3: insulin-like growth factor binding protein-3; PMT: pigmentation; MST: menstruation.

We computed the feature importance score for all the 19 variables to identify important features used by the models. The importance of each feature calculated by the models is plotted in Figure 3. In both models, the most important predictive variable was LH level, followed by IGF-I and FSH levels. The fourth most important feature for the random forest model was height, which ranked fifth in the XGBoost model. Prolactin is the fourth most important feature of XGBoost, but it contributed only a little to random forest. These data suggest that different machine learning algorithms attach importance to different combination lists of variables, although they yield similar predictive performance.

In order to further verify the importance of the top 3 features, we constructed XGBoost models with these features individually or in combination (Table 2). As expected, the models using one, two, or three features had poorer performances than the models using all features. The results showed that the higher the feature ranked, the better the corresponding model performed. Interestingly, LH alone or together with IGF-I and FSH is sufficient to predict a response to the GnRHa test with a fairly good performance and an AUC between 0.84 and 0.86. These data support the results from the feature importance calculations.

### Local Interpretable Model-Agnostic Explanations for Interpretation

**Figure 4:**
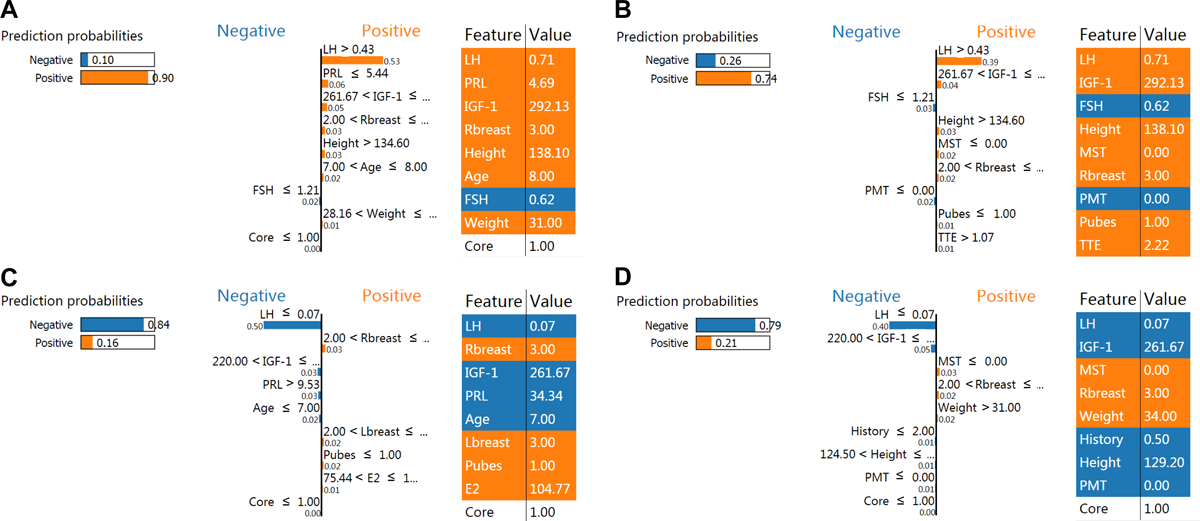
Results of LIME with XGBoost and Random Forest classifiers applied to one positive (A, B) and one negative (C, D) instance. The left sides are for XGBoost, and the right for Random Forest. Blue color is for the negative instance and orange is for the positive instance. The first column represents the prediction probabilities of negative and positive results achieved from classifiers. The second column shows the features’ contributions to the probability. Only the top nine features are displayed for clarity. The third column displays the original data values. LIME: local interpretable model-agnostic explanations; XGBoost: extreme gradient boosting; LH: luteinizing hormone; IGF-I: insulin-like growth factor-I; FSH: follicle-stimulation hormone; PRL: prolactin; GH: growth hormone; E2: estradiol; BMI: body mass index; TTE: testosterone; Rbreast: right breast; Lbreast: left breast; IGFBP-3: insulin-like growth factor binding protein-3; PMT: pigmentation; MST: menstruation; PMT: pigmentation.

A representative set of 200 samples, which accounted for more than 50% of the test set and were enough to be the budget size of individual instances to understand a model, were selected with the submodular pick method [27] for the 19-variable models. LIME was then applied to investigate feature contributions for each prediction. Results with top 9 features are presented in Figure 4 for one positive sample and one negative sample (more representative samples can be seen in Multimedia Appendix 1). In Figure 4, XGBoost predicts an instance where CPP positively responds to the GnRHa test with a probability of 90%. Only the feature growth hormone supports the negative prediction, whereas LH, prolactin, IGF-I, and information about body development support the positive prediction. This makes sense in the clinical diagnosis of CPP and reveals that we can trust our prediction models to a certain extent. In Figure 4, left and right breast at Tanner stage 3, FSH level > 2 IU/L, and several other features support the positive prediction with a probability of 16%. LH level of 0.07 IU/L, prolactin level > 9.53 ng/mL, IGF-I level > 220 ng/mL, and age < 7 years contribute to the negative prediction with a probability of 84%. Similar results are observed in Figure 4.

## Discussion

### Overview

CPP mimics pubertal development ahead of time at an inappropriate chronological age. It requires timely detection and treatment in case of physical and physiological effect on girls. The GnRHa-stimulation test is expensive and time consuming and causes discomfort to patients. Here, we applied machine learning algorithms to multiple clinical variables and built two tree-based ensemble learning classifiers for the prediction of response to the GnRHa-stimulation test. Both the XGBoost and random forest models achieved good performance in distinguishing between positive and negative responses, with the AUC ranging from 0.88 to 0.90, the sensitivity ranging from 77.91% to 77.94%, and the specificity ranging from 84.32% to 87.66%.

### Comparisons with Previous Models

Several previous models focused on determining optimal blood sampling time points or appropriate cut-off values to simplify the stimulation test. Kandemir et al [29] found that a single sample of LH tested at the 40th minute after stimulation with a cut-off of 5 IU/L could yield 98% sensitivity and 100% specificity in the diagnosis of CPP. Yazdani et al [30] showed that an LH concentration > 5 IU/L at 3 hours has optimal sensitivity (83%) and specificity (97%). In the study of Çatlı et al [7], 100% sensitivity and 84% specificity were obtained using a cut-off value > 0.24 for peak LH/FSH ratio in girls. Although these models performed better than our models, they had to be used after stimulation and therefore could not avoid the disadvantages of the GnRH/GnRHa test completely.

Some models used only the basal sex hormone level. Yazdani et al [30] found that a basal LH level of >0.1 IU/L, a basal LH/FSH ratio >1, and basal estradiol level ≥1.5 ng/dL in girls have low sensitivity (10%-67%) but excellent specificity (94%-100%). Çatlı et al [7] also reported models with the basal FSH or LH levels and achieved a sensitivity of 71% and a specificity of 68% or 64%. Pasternak et al [3] reported that basal LH levels ≤ 0.1 IU/L were sufficient to rule out positive response to the GnRH test with a specificity of 94% but a sensitivity of only 64% in girls. In another model [4], the basal LH level with a cut-off value of 0.35 IU/L was associated with a sensitivity of 63.96% and a specificity of 76.3% based on the ROC with an AUC of 0.77. These results varied a lot due to the different settings and sample sizes. In this study, our models showed better performance with more features before stimulation and a larger homogeneous population, which is the largest population in such a study to our knowledge.

### Predictive Features

Based on our machine learning models, basal LH, IGF-I, and FSH levels are predictive factors with top ranks for the feature importance in both models. Previous studies have demonstrated that the measurement of LH could be better than that of other sex hormones for initial evaluation of suspected puberty [3,8]. In our study, the LH level ranked first and was much more important than other variables. Besides LH, another indicator monitored in the stimulation test, FSH, was also selected by the models as the third most–important variable. Obviously, LH and FSH are important to CPP because they are biomarkers of the hypothalamic-pituitary-gonadal axis activation, which is the essence of CPP. IGF-I, which is the second most important variable in our models, is reportedly involved in GnRH regulation [31,32] and is increasingly expressed in girls with CPP [9,5]. Animal studies showed that the IGF-I signaling pathways play important roles in the timing of puberty in girls [32]. Although IGF-I has not been considered in previous models, our study suggests that IGF-I may be a valuable marker for diagnosing CPP.

Several studies [12,33,34] suggested that image reports like pelvic ultrasound and radiography of the hand have adjunct diagnostic values in CPP diagnosis but provide no reliable differentiation alone. Here, we found that adding features from the image reports improved the prediction results. Performance of models built based on 1757 samples was better than that based on 436 samples, suggesting a sample size effect. Interestingly, in the case of 436 samples with additional six image variables, the aforementioned sample size effect was balanced. This suggests that more samples with image features will produce better results. Thus, medical image examinations like bone age radiography should be considered before the GnRHa-stimulation test for girls with suspected CPP.

### Interpretations of Models

We noticed that more variables were assigned with a moderate value of feature importance in the XGBoost model than those in the random forest model. This is reasonable when considering the different algorithms the two models used for prediction and importance evaluation. In XGBoost, trees are sequentially built in a boosting manner to enhance the overall performance. The estimates of feature importance are provided explicitly with the frequency that the feature is selected as a tree node from a trained predictive model. In contrast, trees are trained parallelly in a bootstrapping way in random forest to vote for the final decision. The feature importance is estimated implicitly through permuting the feature’s values and calculating the change of the model’s prediction error. Obviously, XGBoost includes each contribution of each feature to each tree into the feature importance, whereas random forest only evaluates each feature globally without specific contributions. It should be noted that different combinations of variables may produce models with similar predictive accuracy, relating to the uncertainty analysis of the solutions in any decision-making problem [35-37]. This is not rare in machine learning models in medicine [38-40]. Moreover, the most important features in the clinic such as basal LH, IGF-I, and FSH levels were all sorted out by both models, demonstrating that they are both reliable and effective in predicting response to the GnRHa test.

In order to provide endocrinology physicians a trustworthy insight into the prediction models, we also used LIME to show each feature’s contribution to predicting probabilities reasonably. The most important features used by the models for individual prediction have been proven to be significant in the clinic [3-5,7,9,10], demonstrating that our models are credible. This will greatly increase the interpretability of the machine learning models and make it convenient for individualized diagnosis in the clinic.

### Limitations

There are some limitations to this study. First, growth velocity is specially related to physical development. Due to the lack of height growth rate and weight growth rate, we did not include growth velocity in our feature set. For further research, we will focus more on medical imaging and growth velocity to identify their diagnostic value with CPP. Second, our work included only girls with suspected CPP from a single center in China. The prediction models in this study may not be suitable for the population in other districts or countries. Third, manual inspection of values extracted through regular expression matching from free text could reduce errors to improve the model performance. However, this adds a considerable amount of work and thus reduces the scalability of the model. We are improving the matching algorithm with the manually inspected data to increase the level of model automation. Finally, features generated from laboratory results are more complete than those extracted from free text, which may affect the rank of feature importance. More efforts are required to enhance the data quality of unstructured features in the future.

### Conclusions

Our study is the first one to apply both machine learning algorithms and the explanation method to the diagnosis of CPP. Our models can predict the response to the stimulation test before injection of GnRHa in girls who are suspected of having CPP and thus may be used as a prescreening tool to help physicians make decisions in conjunction with the GnRHa-stimulation test.

## Appendix

Multimedia Appendix 1 Results of LIME with XGBoost and Random Forest classifiers applied to four positive and four negative instances. LIME: local interpretable model-agnostic explanations.

## Abbreviations

AUC: area under receiver operating characteristic
BMI: body mass index
CPP: central precocious puberty
FSH: follicle-stimulation hormone
GnRH: gonadotropin releasing hormone
GnRHa: gonadotropin releasing hormone analogues
IGF-I: insulin-like growth factor-I
IGFBP-3: insulin-like growth factor binding protein-3
LH: luteinizing hormone
LIME: local interpretable model-agnostic explanations
MCCV: Monte Carlo cross-validation
OOB: out-of-bag
ROC: receiver operating characteristic
SVM: supported vector machines
XGBoost: extreme gradient boosting
