# Supplementary material for: Development of Prediction Models Using Machine Learning Algorithms for Girls with Suspected Central Precocious Puberty: Retrospective Study
Source: JMIR Med Inform. 2019 Feb 12;7(1):e11728. doi: 10.2196/11728 (PMC6390190; doi:10.2196/11728)
Supplement: Multimedia Appendix 1 [file medinform_v7i1e11728_app1.pdf]

Supplementary Figure S1

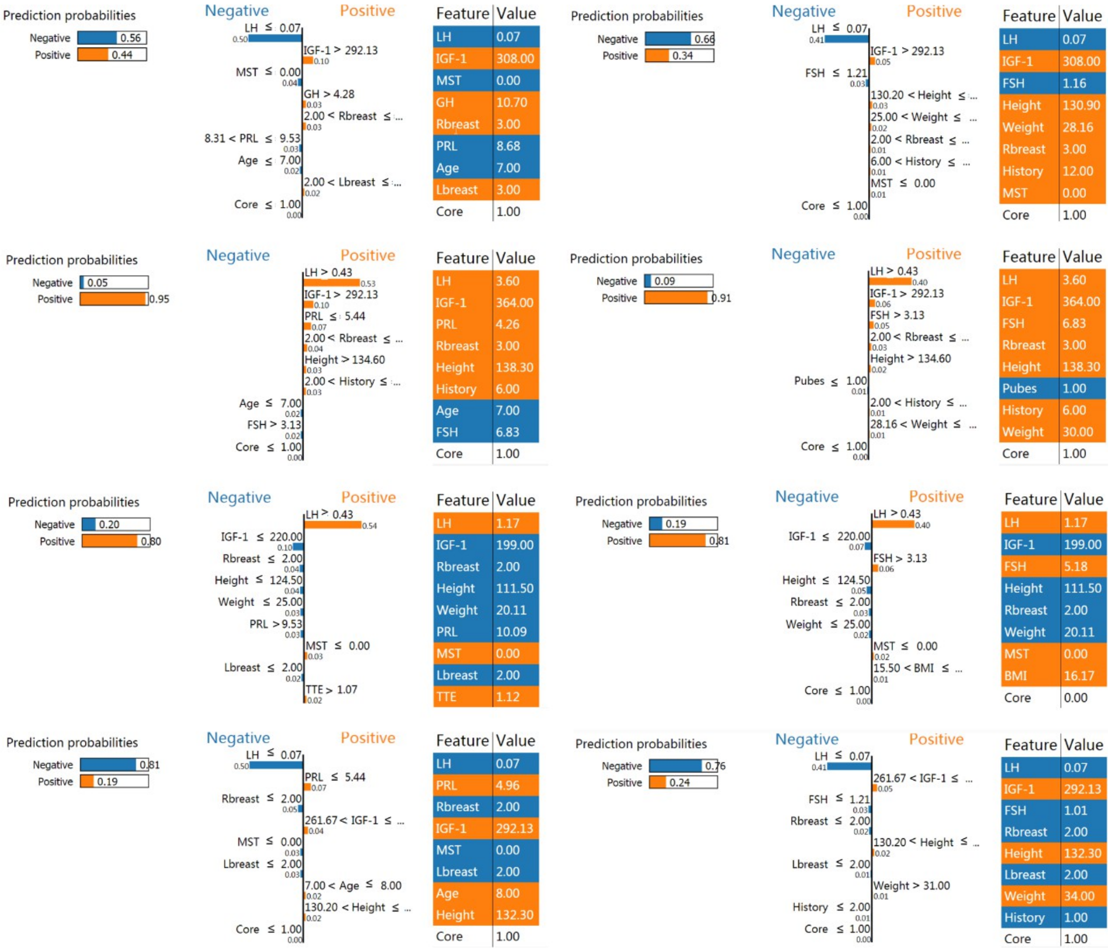

Figure S1. Results of LIME with XGBoost and Random Forest classifiers applied to four positive and four negative instances. The left sides are for XGBoost, and the right for Random Forest. Blue color is for the negative instance and orange is for the positive instance. The first column represents the prediction probabilities of negative and positive results achieved from classifiers. The second column shows the features' contributions to the probability. Only the top nine features are displayed for clarity. The third column displays the original data values. LIME, local interpretable model-agnostic explanations; XGBoost, extreme gradient boosting; LH, luteinizing hormone; IGF-I, insulin-like growth factor-I; FSH, follicle-stimulation hormone; PRL, prolactin; GH, growth hormone; E2, estradiol; BMI, body mass index; TTE, testosterone; Rbreast, right breast; Lbreast, left breast; IGFBP-3, insulin-like growth factor binding protein-3; PMT, pigmentation; MST, menstruation; PMT, pigmentation.
